# Cardiac Screening in a Young Adult Male Leading to Discovery of Post-COVID Myocarditis with Asymptomatic Large Apical Left Ventricular Thrombus

**DOI:** 10.1016/j.case.2021.07.008

**Published:** 2021-08-14

**Authors:** David Munoz, Hamza Malik, Daniel Eickenhorst, Stephen Newman, Cyril Varughese, Farhan Ali

**Affiliations:** a Department of Internal Medicine, Medical City Weatherford, Weatherford, Texas; b Texas College of Osteopathic Medicine, University of North Texas Health Science Center, Fort Worth, Texas; c Department of Radiology, Baylor Scott and White All Saints, Fort Worth, Texas; d Department of Cardiology, Baylor Scott and White All Saints, Fort Worth, Texas

**Keywords:** Myocarditis, Left ventricular thrombus, COVID-19, SARS-CoV-2, Echocardiography

## Abstract

• A unique sequela of COVID-19 is COVID-related ventricular thrombus formation.
• Diagnosis of a ventricular thrombus can only be confirmed through imaging.
• COVID-related myocarditis can ultimately lead to ventricular thrombus formation.
• Post-COVID cardiac screening may reveal sequelae in asymptomatic athletic individuals.

## Graphical abstract

**Figure:**
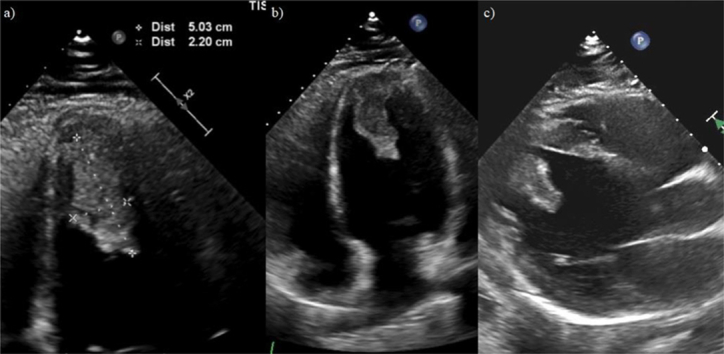

## Introduction

It has now been a year and a half since the start of the COVID-19 pandemic. During that time, our understanding on how the SARS-CoV-2 virus affects humans has grown exponentially. However, despite this ever-growing body of evidence, we still do not fully understand what factors make patients susceptible to post-COVID-19 complications. Patients who have no or only a few risk factors can still experience uncommon sequelae. One of the unique sequelae following a COVID-19 infection has been COVID-related ventricular thrombus formation. To our knowledge, only a handful of cases resulting in ventricular thrombi caused by COVID-19 have been published.^1^, ^2^, ^3^, ^4^, ^5^, ^6^, ^7^ All of the published cases were of relatively older patients (>40 years old). Additionally, another less common cardiovascular complication, with the most recent studies calculating an incidence of about 33%,^8^ is COVID-related myocarditis. In this report, we describe the first case, to our knowledge, of an adult man <20 years old with COVID-related myocarditis leading to a reduced ejection fraction and subsequent ventricular thrombus.

## Case Presentation

A previously healthy 18-year-old Hispanic college American football player was referred to the cardiology clinic in November 2020 due to an elevated conventional troponin I level found during routine clearance for playing. In September, he had a 3-day history of fevers, myalgias, nausea, and vomiting. He was tested for COVID-19 at the time and tested positive; however, following this 3-day symptomatic period, he recovered without any issues and did not require any intervention. Following this recovery, the patient reported being asymptomatic until the week prior to testing, when he began to experience mild fatigue.

**Figure 1:**
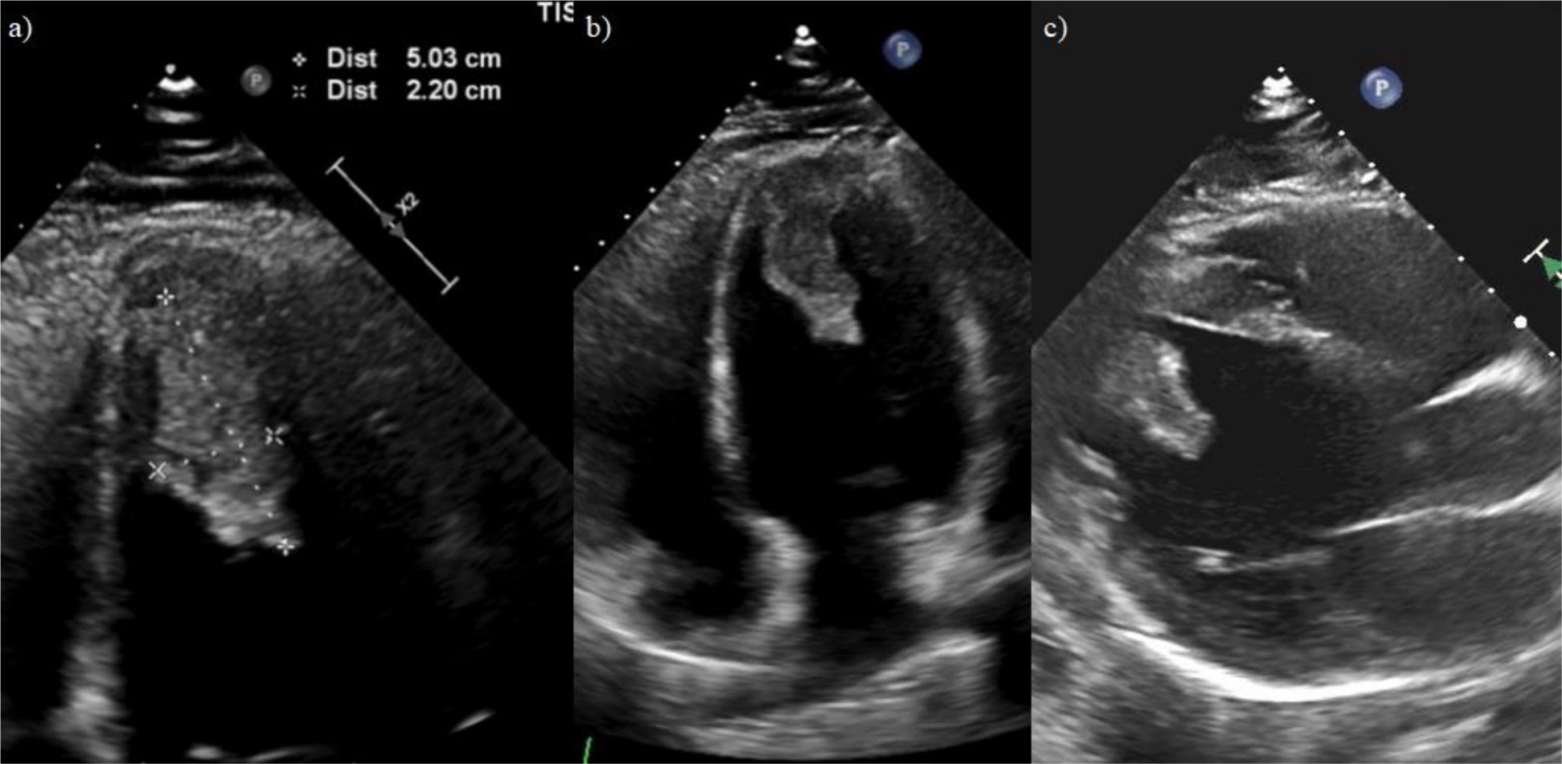
Transthoracic echocardiogram without contrast. **(A)** Zoomed apical four-chamber view demonstrating the LV apical mass with dimensions of 5 × 2.2 cm. **(B)** Apical four-chamber view demonstrating a hyperechoic, mobile, and pedunculated LV apical mass with margins distinct from the endocardial edge and defined contours consistent with thrombus. **(C)** Left parasternal long-axis view demonstrating the LV apical mass extending into the midcavity.

**Figure 2:**
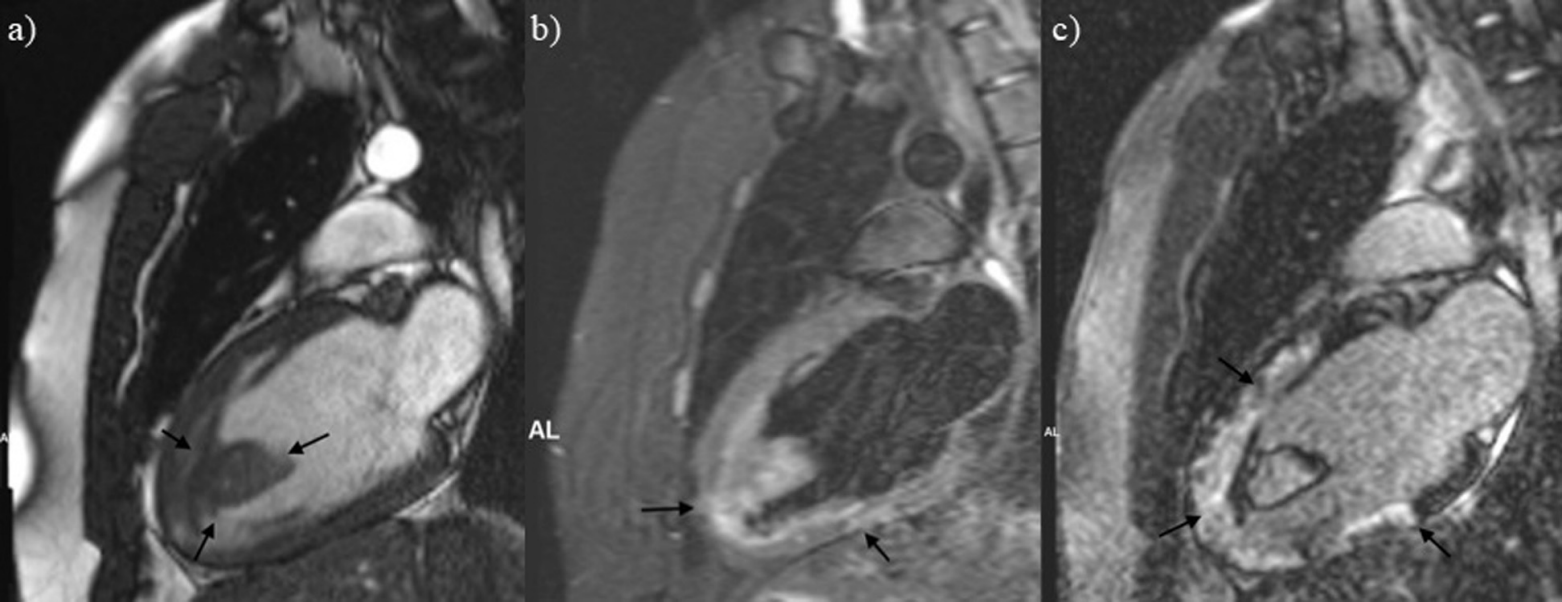
Cardiovascular magnetic resonance imaging two-chamber view. **(A)** Large wall-adherent apical thrombus, which appears isointense to myocardium (*arrows*) on a CINE (TruFISP) image still. **(B)** Apical thrombus, which appears hyperintense to myocardium and with areas of mild patchy edema within the LV apex and inferior wall (*arrows*) on a triple inversion recovery image. **(C)** Large areas of transmural and midmyocardial delayed myocardial enhancement reflecting areas of necrosis/fibrosis (*arrows*) on a delayed postcontrast image.

In the cardiology clinic, the patient's elevated conventional troponin I level in absence of symptoms suggestive of acute coronary syndrome and with a history of recent COVID-19 infection raised concerns of possible myocardial injury. As such, the patient was admitted to the hospital and underwent further imaging. An initial transthoracic echocardiogram was performed that showed decreased systolic left ventricular (LV) function with an LV ejection fraction (LVEF) of 40% and a large 5.0 × 2.2 cm fixed mass located in the apex of the left ventricle with some mobility at the tip. There was no pericardial effusion or significant LV hypertrophy seen to suggest an infiltrative or pericardial disease (Figure 1 and Videos 1 and 2). Lab work revealed a hemoglobin A1c of 5.1%, a conventional troponin I of 0.12 ng/mL (0.00-0.05 ng/mL), a d-dimer of 0.39 μg/mL (<0.5 μg/mL), a B-natriuretic peptide of 34 ng/L (5-100 ng/L), and a thyroid-stimulating hormone of 3.95 mIU/mL (0.36-3.74 mIU/mL). The cardiac magnetic resonance imaging showed a dilated left ventricle with moderately reduced systolic function with global hypokinesis and a calculated LVEF of 38%. Additionally, it further demonstrated areas of patchy LV myocardial edema with associated areas of midmyocardial and subepicardial delayed enhancement. Given the patient's clinical picture, the cardiac magnetic resonance imaging findings were most consistent with myocarditis based on the revised Lake Louise criteria (late gadolinium enhancement on T1-based imaging and visible myocardial edema on T2-based imaging). The LV mass appeared to be originating from the LV apex and demonstrated heterogeneous signal intensity without appreciable enhancement. Given the location of the mass and reduced systolic function, the mass was favored to represent large LV thrombus (Figure 2).

**Figure 3:**
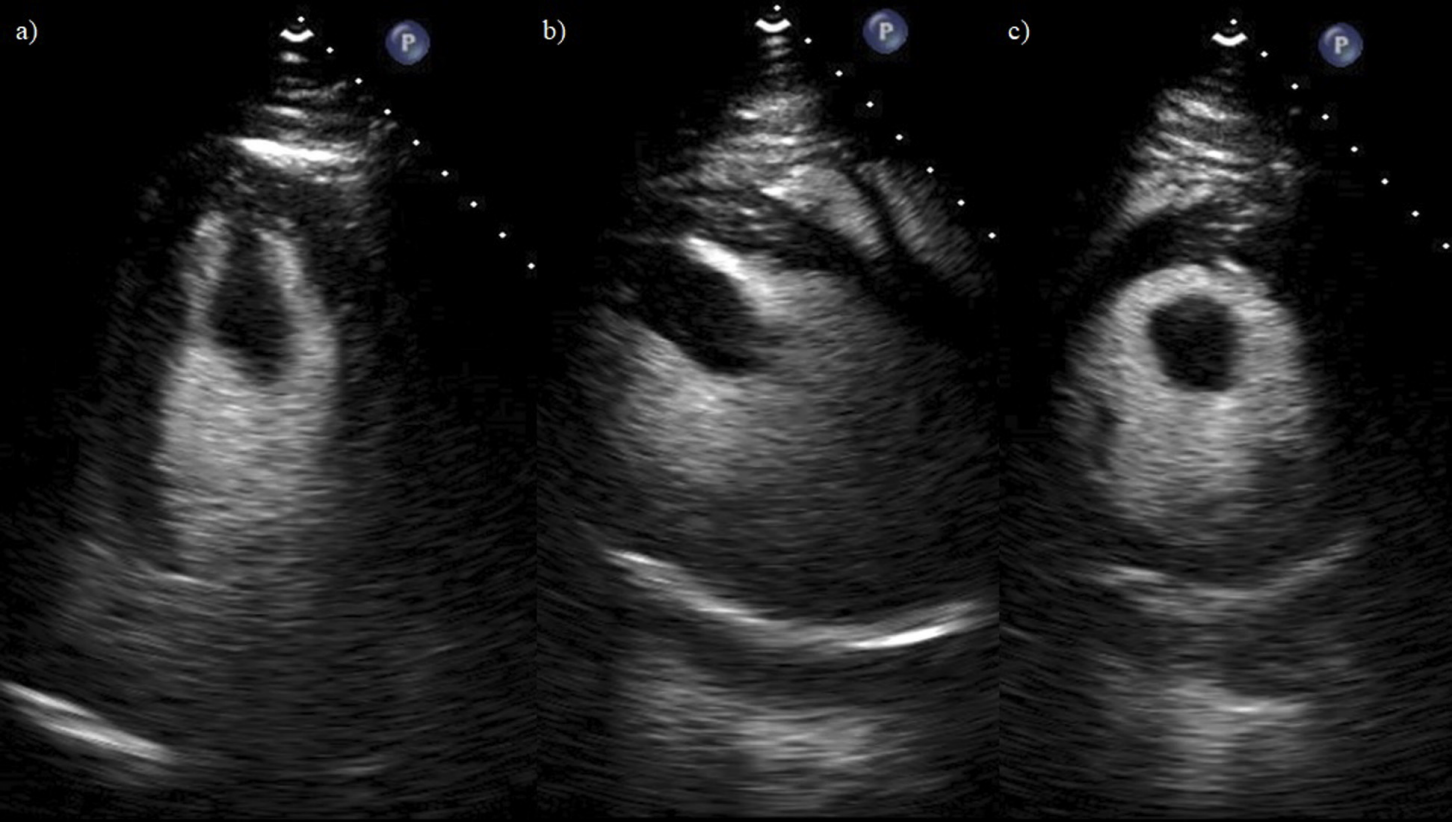
Transthoracic echocardiogram with perflutren lipid microsphere contrast. **(A)** Perflutren lipid microsphere contrast-enhanced apical four-chamber view of the hyperechoic, pedunculated, and mobile apical mass. **(B)** Perflutren lipid microsphere contrast-enhanced left parasternal long-axis view of LV apical mass. **(C)** Perflutren lipid microsphere contrast-enhanced left parasternal short-axis view of LV apical mass.

Upon admission, the patient was started on a continuous intravenous heparin drip at 18 units/kg/hour and warfarin 7.5 mg once daily. Standard heart failure therapy was also initiated in the form of losartan, carvedilol, and spironolactone. A repeat transthoracic echocardiogram with perflutren lipid microsphere contrast was then performed, which showed enhancement of the LV apical mass with an LVEF of 40%-45% with moderate hypokinesis of the apex (Figure 3 and Videos 3-5) The intravenous heparin drip was continued until the patient reached an international normalized ratio (INR) of 2.7. The patient had genetic screening performed of up to 106 genes including transthyretin amyloid, LV noncompaction, arrhythmogenic right ventricular cardiomyopathy, and other various hereditary cardiomyopathies. The genetic screening showed no findings consistent with the patient's presentation. The patient was then discharged from the hospital after the sixth day of admission on heart failure medications and warfarin, the latter of which had the goal of maintaining the patient's INR between 2.5 and 3.

**Figure 4:**
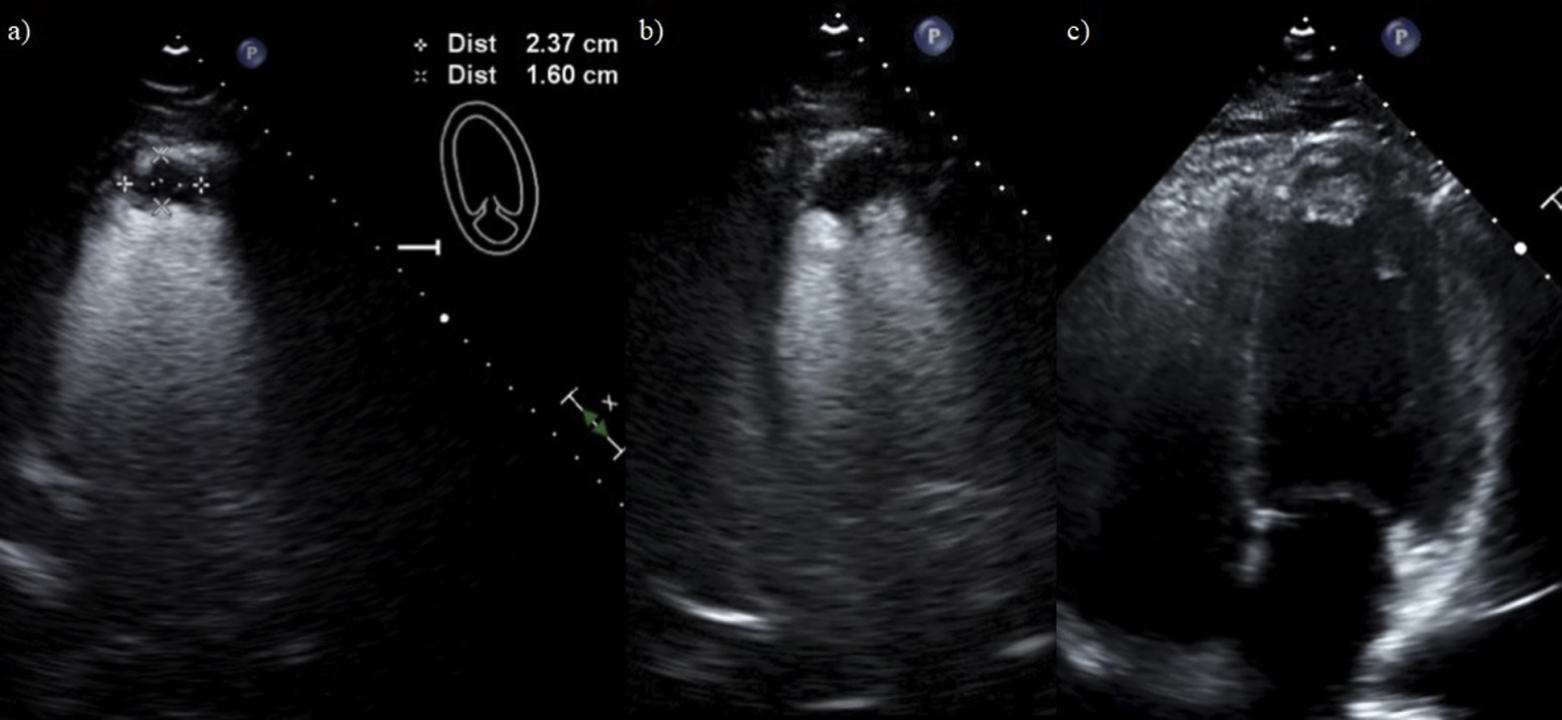
Five-month follow-up transthoracic echocardiogram with and without perflutren lipid microsphere contrast. **(A)** Perflutren lipid microsphere contrast-enhanced apical two-chamber view of the LV apical mass on follow-up imaging 5 months from the initial images demonstrating reduced size of the mass. **(B)** Perflutren lipid microsphere contrast-enhanced apical four-chamber view of the LV apical mass on follow-up imaging demonstrating reduced size of the mass. **(C)** Apical four-chamber noncontrast grayscale view of the LV apical mass on follow-up imaging demonstrating reduced size of the mass.

The patient continued to have regular follow-ups as an outpatient, both to monitor his INR ratio and to monitor the progression of the LV thrombus. Initially, the patient was noted to be doing well, with progressive reduction in the LV thrombus size. However, a few months after his initial diagnosis, the patient started to become nonadherent with his medications. Repeat transthoracic echocardiograms with and without perflutren lipid microsphere contrast were then once again performed, which showed a decreased LVEF of 35%-39% with moderate global hypokinesis and an apical mass with dimensions of 2.37 × 1.6 cm (Figure 4 and Videos 6 and 7). The patient was educated on the need to continue therapy. The patient was receptive to the feedback and began to once again be compliant with his medications.

## Discussion

At the time of writing, it has been one year since the SARS-CoV-2 virus was declared a pandemic by the World Health Organization. Thus, our knowledge of the virus has increased significantly from when the first cases were reported in Wuhan, China. Nevertheless, there are still several aspects of the virus that have not been as well researched.

This case highlights one of these aspects that requires further research: a deeper understanding of the significance of the presence of specific risk factors that contribute to making a patient more susceptible to significant sequelae following a COVID-19 infection. One meta-analysis analyzing the risk factors implicated in a more severe clinical course, and thus a poorer prognosis, showed that factors such as old age, male gender, belonging to a nonwhite ethnic group, and comorbidities such as hypertension, obesity, diabetes mellitus, and cardiovascular disease were significant contributors.^9^

In our case, the patient did have some of the risk factors described in the study such as being male, of Hispanic origin, and with a body mass index of 35.72, consistent with class II obesity. However, with regards to his body mass index, it is important to note that the patient's abdomen-to-waist circumference ratio was 0.9, which per World Health Organization guidelines suggest a healthy, albeit borderline, ratio.^10^ Furthermore, being an athlete likely also was a contributing factor. It has been reported that in patients who go on to develop post-COVID myocarditis, exercise is likely to worsen cardiac dysfunction. This dysfunction is attributed both to accelerated viral replication and increased inflammation and cellular apoptosis.^11^ As of now, however, it is unclear whether these factors by themselves are significant enough to fully explain why a previously healthy 18-year-old athlete developed such severe sequelae, and more research into this topic is needed. Nevertheless, based on available data, it would be reasonable to state that highly physically active individuals could benefit from post-COVID infection cardiac screening if they are asymptomatic or minimally symptomatic as this population has a discernible risk for the development of significant cardiac complications.

## Conclusion

To our knowledge, this is the first reported case of an adult man <20 years old with COVID-related myocarditis leading to a reduced ejection fraction and subsequent ventricular thrombus formation. We believe the focus of clinical care will soon require a shift toward early detection of sequelae of COVID-19, especially in highly active younger individuals where patients are less likely to show overt symptoms.

## Supplementary Data

Video 1 Initial noncontrast transthoracic echocardiogram apical four-chamber view demonstrating a hyperechoic, mobile, and pedunculated LV apical mass with margins distinct from the endocardial edge and defined contours consistent with thrombus.

Video 2 Initial noncontrast transthoracic echocardiogram left parasternal long-axis view demonstrating the LV apical mass extending into the midcavity.

Video 3 Initial contrast-enhanced transthoracic echocardiogram apical four-chamber view demonstrating the hyperechoic, pedunculated, and mobile apical mass.

Video 4 Initial contrast-enhanced transthoracic echocardiogram left parasternal long-axis view demonstrating the LV apical mass.

Video 5 Initial contrast-enhanced transthoracic echocardiogram left parasternal short-axis view demonstrating the LV apical mass.

Video 6 Follow-up contrast-enhanced transthoracic echocardiogram apical four-chamber view of the LV apical mass on follow-up imaging 5 months from the initial images demonstrating reduced size of the mass.

Video 7 Follow up noncontrast transthoracic echocardiogram apical four-chamber view of the LV apical mass on follow-up imaging 5 months from the initial images demonstrating reduced size of the mass.
